# Divergent cellular responses to sciatic nerve crush versus transection: bilateral MYCN induction and distinct DRG neuronal responses

**DOI:** 10.3389/fncel.2026.1922426

**Published:** 2026-09-28

**Authors:** Shilian Zhou, Ziyan Huang, Zhongyuan Lin, Jingwei Chen, Xinyu Zhang, Diya Wang, Haihua Shu, Fang Ye

**Affiliations:** 1 Guangdong Cardiovascular Institute, Guangdong Provincial People's Hospital, Guangdong Academy of Medical Sciences, Guangzhou, China; 2 Department of Anesthesiology, First Affiliated Hospital, Sun Yat-sen University, Guangzhou, China; 3 Dermatology Hospital, Southern Medical University, Guangzhou, China; 4 The First School of Clinical Medicine, Southern Medical University, Guangzhou, China; 5 Department of Philosophy, Sun Yat-sen University, Guangzhou, China; 6 Key Laboratory of Biomedical Information Engineering of Ministry of Education, Department of Biomedical Engineering, School of Life Science and Technology, Xi'an Jiaotong University, Xi'an, China; 7 Department of Anesthesiology, Guangdong Provincial People's Hospital, Guangdong Academy of Medical Sciences, Southern Medical University, Guangzhou, China

**Keywords:** ATF3, bilateral spinal response, MYCN, nerve regeneration, peripheral nerve injury, sciatic nerve axotomy, sciatic nerve crush, sensory dysfunction

## Abstract

**Introduction:**

Peripheral nerve injuries can produce regenerative or persistent-disconnection outcomes depending on the injury modality, but the underlying cellular responses and their effects on the central nervous system remain incompletely defined.

**Methods:**

We compared sciatic nerve crush (SNC; axonotmesis) and sciatic nerve transection (SNT; neurotmesis) in wild-type, AdvCre;Ai14, and MYCN-eGFP reporter mice. Longitudinal assessments included functional recovery, neuronal injury responses marked by ATF3, IB4-positive sensory-neuron responses, dorsal-horn microglial activation, and spinal MYCN-eGFP signal.

**Results:**

SNC was associated with partial, measure-specific functional recovery, a transient and self-limited ATF3 response, and a limited reduction in IB4-positive neuronal profiles. In contrast, SNT produced persistent axotomy-associated neuronal stress, a reduction in IB4-positive neuronal profiles, and more protracted dorsal-horn microglial activation. MYCN-eGFP intensity was significantly increased above sham levels bilaterally in spinal ventral-horn neurons after both SNC and SNT at postoperative day 7 (POD7), with the greatest acute amplitude observed after SNC. From POD7 to POD14, MYCN-eGFP intensity declined by more than 90% after SNC but by approximately 60% after SNT, indicating distinct temporal patterns between the two injury modalities.

**Discussion:**

SNC and SNT engage overlapping but quantitatively and temporally distinct cellular programs, including modality-specific dorsal root ganglion neuronal responses associated with functional recovery. These findings refine the interpretation of two widely used peripheral nerve injury models and identify bilateral spinal MYCN-eGFP dynamics as a candidate cellular readout for future mechanistic investigation.

## Highlights

- SNC and SNT elicit overlapping but temporally and quantitatively distinct cellular programs in DRG and spinal cord, rather than categorically different “repair versus pathology” trajectories.
- SNT, but not SNC, is associated with persistent ATF3 expression in DRG and a sustained reduction in IB4-positive neuronal labeling, indicating a differential response of this neuronal phenotype.
- Peripheral nerve injury triggers bilateral MYCN-eGFP induction in spinal ventral-horn neurons, with distinct temporal patterns between SNC and SNT.
- Iba1-positive microglial responses in the spinal dorsal horn are more sustained after SNT, with qualitative Cx3cr1/CD11b morphological changes at POD7.
- These findings support SNC and SNT as complementary models whose divergent DRG and spinal cord signatures merit further mechanistic dissection.

## 1 Introduction

Peripheral nerve injury (PNI) represents a significant clinical challenge, often leading to chronic pain, loss of motor function, and permanent disability. Animal models of PNI are indispensable for dissecting the complex pathophysiology of nerve degeneration and regeneration, and for developing effective therapeutic strategies (Geuna, 2015).

Well-characterized animal models remain a vital and cost-effective tool for translating findings from the bench to clinical application in surgical and regenerative medicine more broadly (Choudhary et al., 2025).

Among these, the rodent sciatic nerve injury model stands as a cornerstone of preclinical research, providing a well-characterized platform to investigate the molecular and cellular responses to axonal damage at the level of the peripheral nerve, the dorsal root ganglia (DRG), and the central nervous system (CNS) (Li et al., 2022).

Two of the most widely employed variants of this model are the sciatic nerve crush (SNC), which induces axonotmesis, and the sciatic nerve transection (SNT), a neurotmesis. In SNC, the axonal continuity is disrupted while the surrounding endoneurial sheaths remain intact. This preservation of structural integrity provides a natural scaffold that guides regenerating axons back to their denervated targets, making it a robust model for studying the innate mechanisms of successful nerve repair (George et al., 2004; He et al., 2023). Conversely, SNT severs all neural and supporting elements, creating a physical gap that precludes spontaneous regeneration. This model more accurately reflects the pathology of severe, complete nerve injuries and is critical for evaluating surgical interventions and advanced bioengineering approaches to nerve repair (Chen et al., 2025).

We emphasize that, because our SNT model additionally involves resection of a 2–4 mm nerve segment with no surgical repair (Section 2.2), SNC and SNT in this study differ not only in the severity of the initiating lesion but also in nerve continuity, regenerative pathway, and reinnervation potential. We therefore treat the two models as a regeneration-permissive injury versus a persistent-disconnection injury, rather than as two points on a single injury-severity scale; where we use “injury severity” as shorthand elsewhere in the manuscript, it refers to this broader set of differences rather than a strictly graded, single-axis measure. A transection model followed by immediate end-to-end repair would be needed to isolate injury severity more directly from nerve continuity, and we identify this as an important direction for future work (Section 4).

Despite the widespread use of both SNC and SNT models, a gap persists in our understanding of how the initial injury modality shapes the subsequent cellular response of repair or degeneration. The literature has largely focused on comparing differential functional outcomes, leaving the upstream cellular triggers and downstream spinal cord consequences poorly integrated. Specifically, two questions remain unanswered: First, how does the loss of structural continuity in SNT reshape the response of injured neurons, shifting them from a state of transient activation (regeneration) toward one of chronic stress (degeneration)? Second, how do differences in injury modality and regenerative potential influence the nature and extent of spinal cord responses, potentially unmasking latent programs for reorganization that are less apparent when local repair succeeds?

We hypothesized that different injury patterns (axonotmesis vs. neurotmesis) trigger distinct retrograde signaling cascades, leading to differential spatiotemporal expression of key transcription factors, such as the canonical injury marker ATF3 and the neurogenesis-associated factor MYCN. These divergent signatures, in turn, would relate not only to the local regenerative response but also to the fate of specific neuronal subpopulations and to plastic changes within the spinal cord. Recent studies have highlighted the role of specific transcription factors in reactivating developmental gene programs to promote axonal regrowth, providing a conceptual framework for our findings on MYCN-associated cellular reprogramming (Zhang et al., 2023; Wu et al., 2024). In this study, we aimed to test this hypothesis by performing a comprehensive, multi-level comparative analysis of SNC and SNT models, leveraging the power of AdvCre; Ai14 (Meltzer et al., 2023) and MYCN-eGFP (Chen and Guan, 2021) transgenic mice to: (1) compare the spatiotemporal expression patterns of ATF3 and MYCN in the DRG and spinal cord, with particular attention to neuronal-subtype-specific responses; (2) assess the differential neuroinflammatory response in the spinal dorsal horn; and (3) correlate these cellular signatures with the time course of sensory and motor functional restitution. By providing this integrated view, we aim to define the distinct cellular signatures of crush and transection injuries, refine the interpretation of these models, and characterize bilateral spinal MYCN dynamics as a candidate readout of differential injury responses for future mechanistic and therapeutic investigation.

## 2 Materials and methods

### 2.1 Experimental animals

All animal procedures were approved by the Institutional Animal Care and Use Committee of Sun Yat-sen University (protocol code 伦审动 (Ref. 2023)365号, approved January 4, 2024). Adult (8–10-week-old) male and female wild-type C57BL/6 J mice, AdvCre; Ai14 (Advillin-Cre line: JAX Stock No. 032536) mice, and MYCN-eGFP transgenic mice on a C57BL/6 J background were used in this study.

Mice were housed under a 12-h light/dark cycle with ad libitum access to food and water, with 6 mice housed per cage. All efforts were made to minimize animal suffering and reduce the number of animals used. A total of 120 mice were used across three experimental groups: Sham (*n* = 36), SNC (*n* = 42), and SNT (*n* = 42), with equal numbers of males and females in each group. Group sizes are not perfectly balanced across Sham/SNC/SNT because each genotype was allocated and powered independently (wild-type behavioral cohort: Sham *n* = 6, SNC *n* = 8, SNT *n* = 8 per time point; AdvCre; Ai14 and MYCN-eGFP reporter cohorts: n = 3/group/time point each); Supplementary Table S1 provides the complete breakdown of animal allocation by genotype, injury group, time point, and downstream assay, and supersedes the totals in this paragraph in case of any discrepancy.

Throughout this study, “Sham” refers to animals that underwent identical anesthesia and surgical exposure of the sciatic nerve without crush or transection (Sections 2.2–2.3); no additional treatment-naïve (unoperated) animals were used, and no naive baseline is reported anywhere in this manuscript. Sham tissue was quantified as the baseline control for the MYCN-eGFP analysis (Figure 1B); for the DRG and dorsal-horn endpoints (ATF3, IB4, and Iba1), the contralateral, unoperated side of each SNC/SNT animal served as the matched internal control (Sections 3.3–3.5). We note in the Discussion (Section 4) that this contralateral-as-control assumption is not absolute, given the bilateral MYCN response we also report.

**Figure 1:**
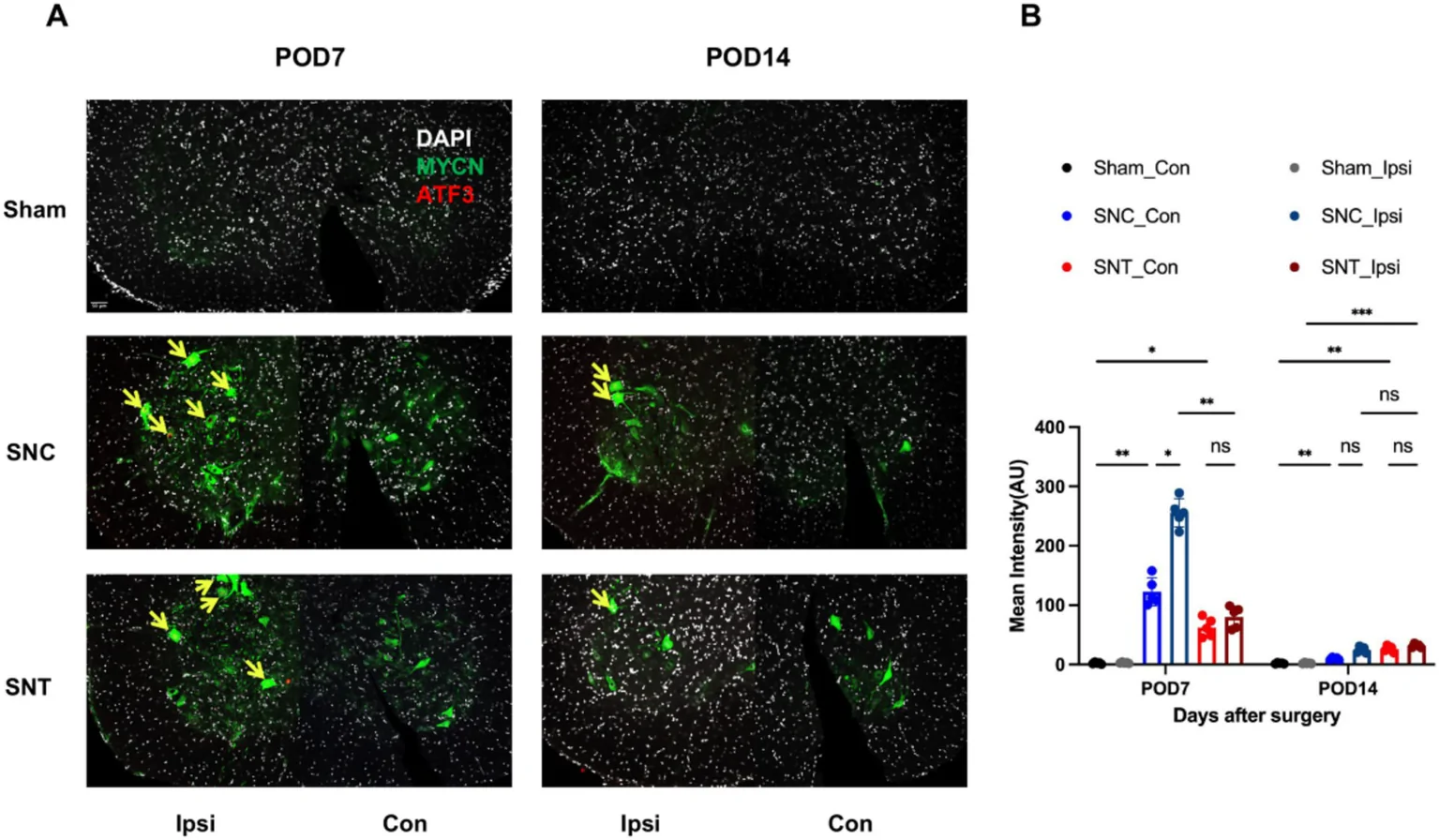
MYCN-eGFP and ATF3 signals in spinal ventral-horn neurons following nerve injury. **(A)** Representative immunofluorescence images of spinal ventral horn stained for DAPI, MYCN-eGFP (green), ATF3 (red), and merge (co-localization indicated by yellow arrow), for Sham, SNC, and SNT groups, ipsilateral (Ipsi) and contralateral (Con), at POD7 and POD14. Scale bar = 50 μm. **(B)** Quantification of MYCN-eGFP mean fluorescence intensity (AU) for all six Group x Side combinations at POD7 and POD14. Mean ± SD, n = 3 animals/group/time point. Two-way ANOVA with Sidak-corrected multiple comparisons; *p < 0.05, **p < 0.01, ***p < 0.001; ns = not significant. Exact statistics (t, df, adjusted p, 95% CI) in Supplementary Table S3 and in Results Section 3.4.

The overall experimental design and timeline of this study are illustrated in Figure 2. Animals were assigned to one of three terminal time-point cohorts (POD7, POD14, or POD28), each an independent, separately allocated set of mice; each animal was followed behaviorally from baseline up to its own assigned endpoint and then euthanized for tissue collection at that time point (for example, a POD28-cohort animal was tested at baseline, POD7, POD14, and POD28 before euthanasia; a POD7-cohort animal was tested only at baseline and POD7 before euthanasia). Behavioral measurements are therefore repeated (longitudinal) within each cohort up to its own endpoint, but the POD7, POD14, and POD28 histological datasets shown in Figures 1, 3, 4, 5, 6 come from distinct, separately euthanized animals rather than serial biopsies of a single surviving cohort; this cohort structure is reflected in the repeated-measures model described in Section 2.7 and in the per-time-point n’s reported in Supplementary Table S1.

**Figure 2:**
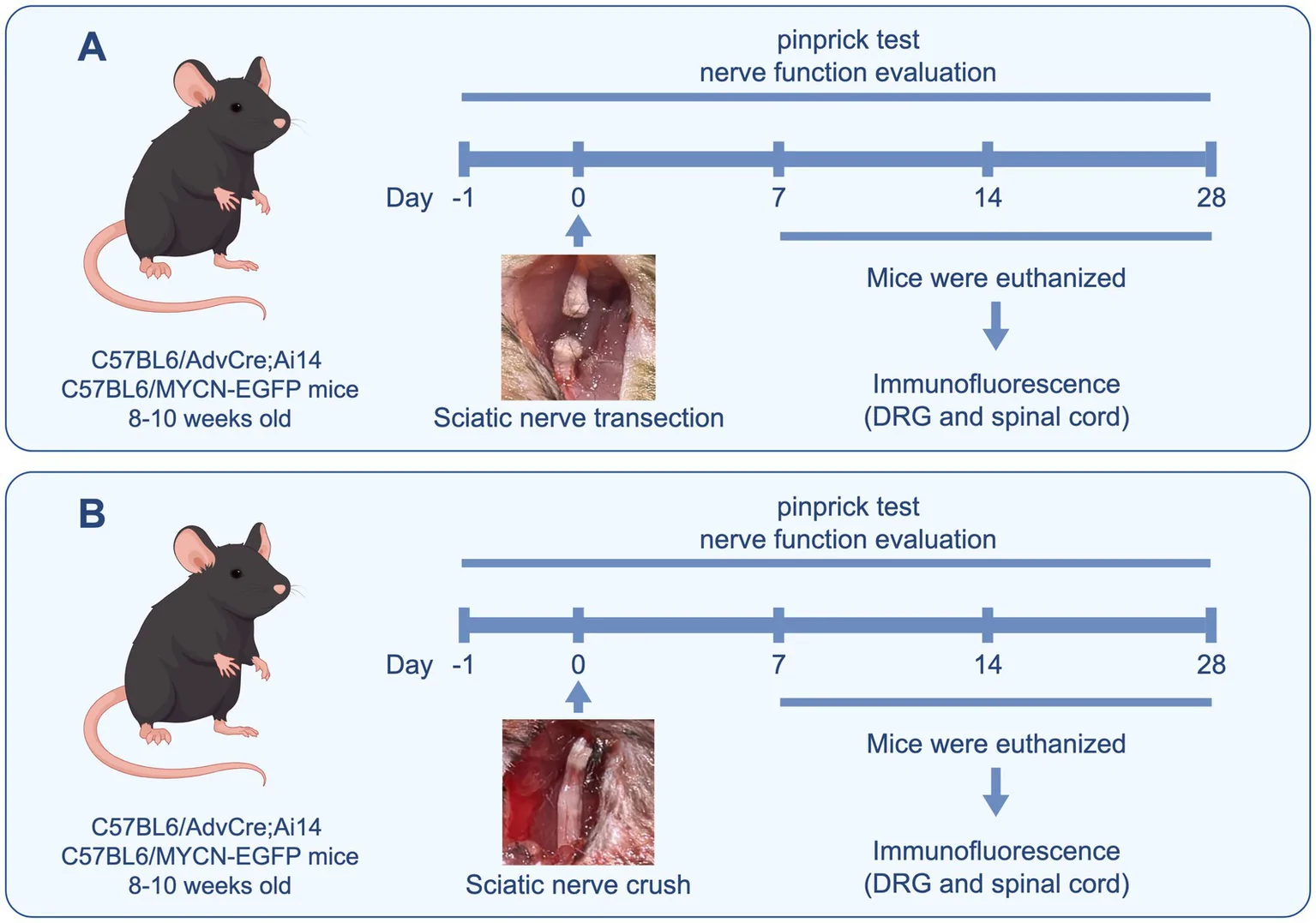
Experimental timeline. Schematic representation of the experimental timeline showing the time points for sciatic nerve injury (SNC or SNT), pinprick test, nerve function evaluation (walking track analysis), and euthanasia for tissue collection at 7, 14, and 28 days post-injury. n per group/time point as specified in Supplementary Table S1.

**Figure 3:**
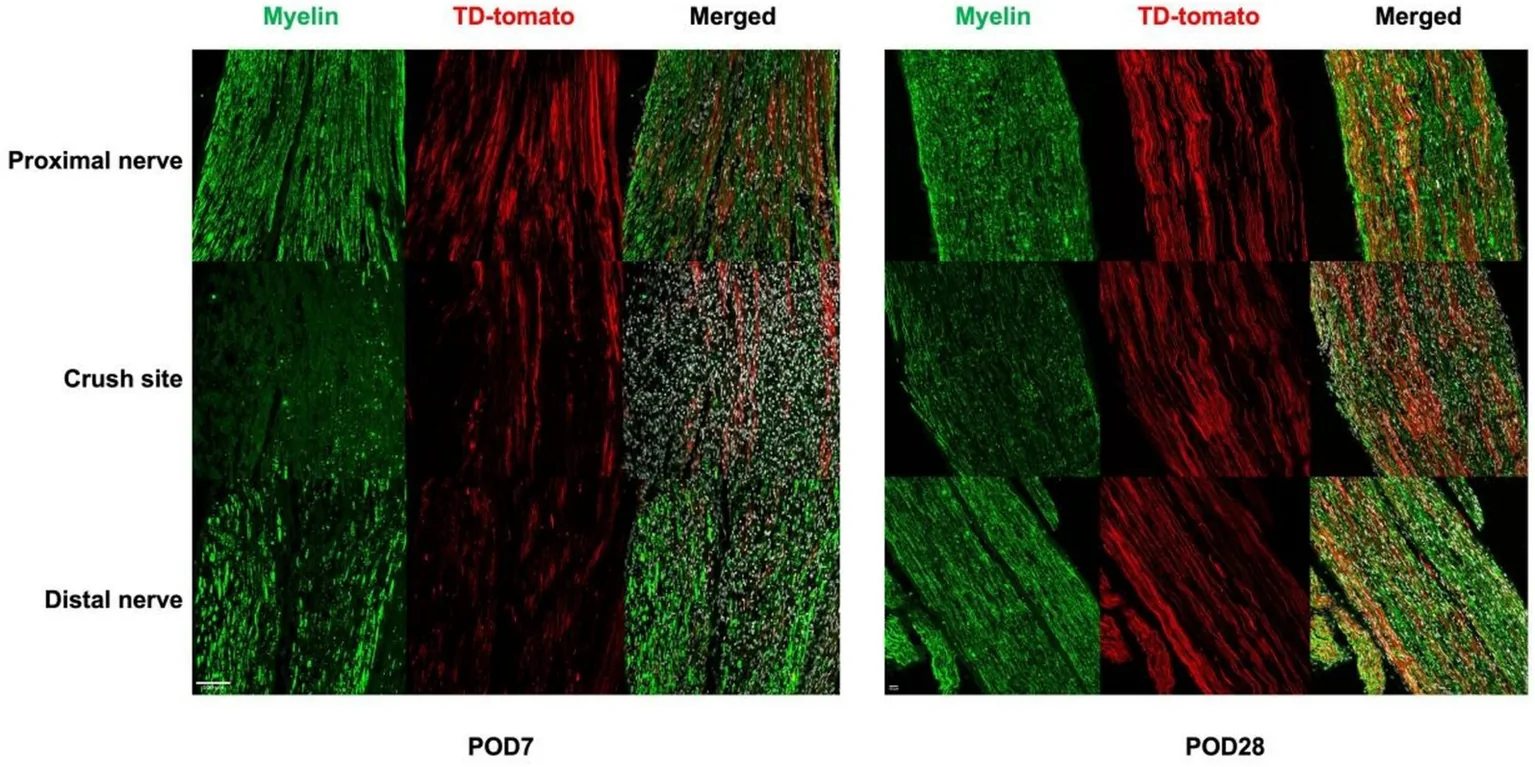
SNC-induced axonal disruption and subsequent restoration of distal axonal labeling in AdvCre; Ai14 mice. Representative confocal images of longitudinal sciatic nerve sections 7 and 28 days post-SNC immunostained for myelin (green) and showing endogenous tdTomato fluorescence (red) in axons. Regions of interest include the uninjured proximal segment, the crush site, and the distal segment. Scale bar = 100 μm.

**Figure 4:**
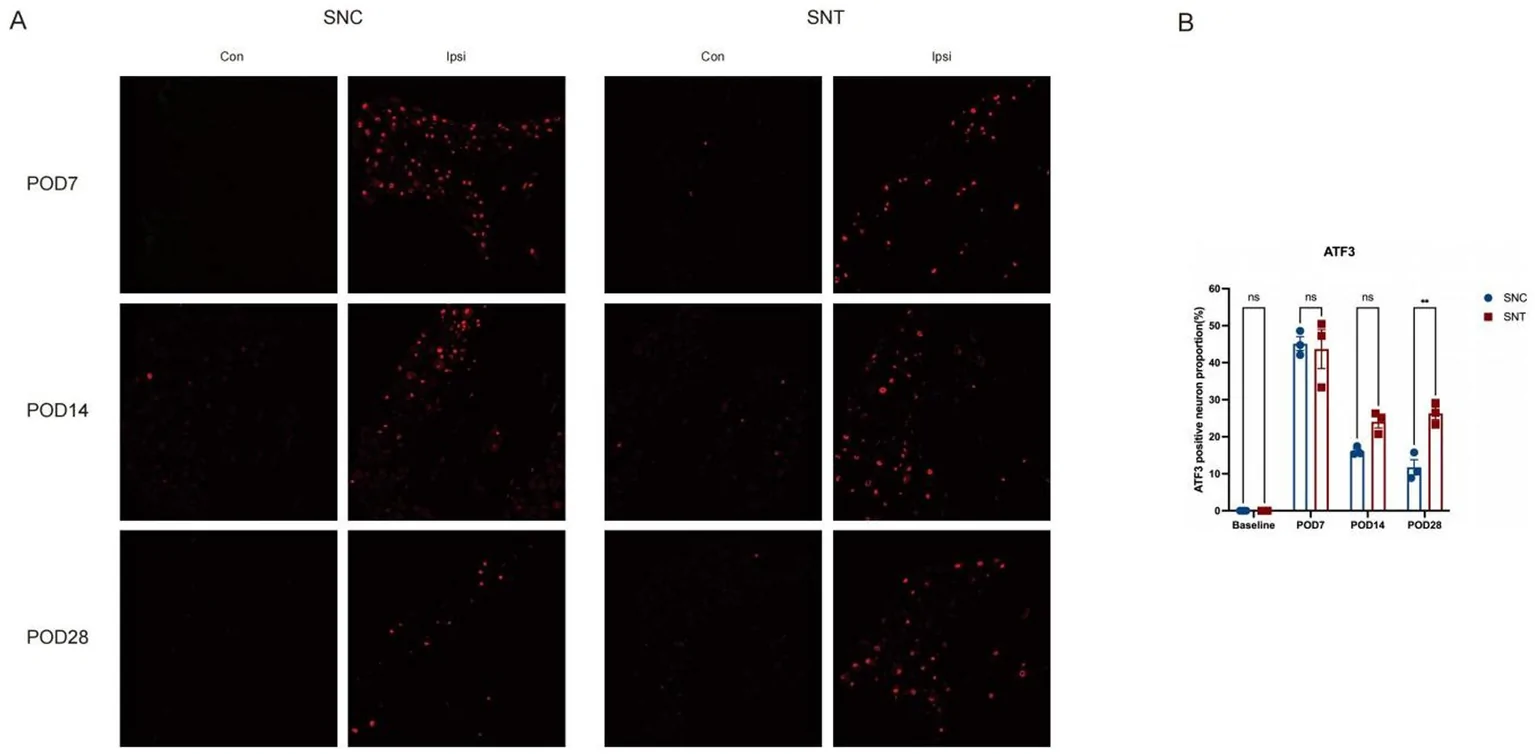
ATF3 expression in DRG neurons following SNC and SNT procedures. **(A)** Representative immunofluorescence images showing ATF3 (red) in contralateral (Con) and ipsilateral (Ipsi) L4-L6 DRG at POD7, 14, and 28. DAPI (blue). Scale bar = 20 μm. **(B)** Quantification of ATF3-positive cells as a percentage of total profiles per section (not an ipsi/contra ratio). Mean ± SD. n = 3 mice per group per time point. **p = 0.0014 vs. SNC at POD28 (corrected; Supplementary Table S3).

**Figure 5:**
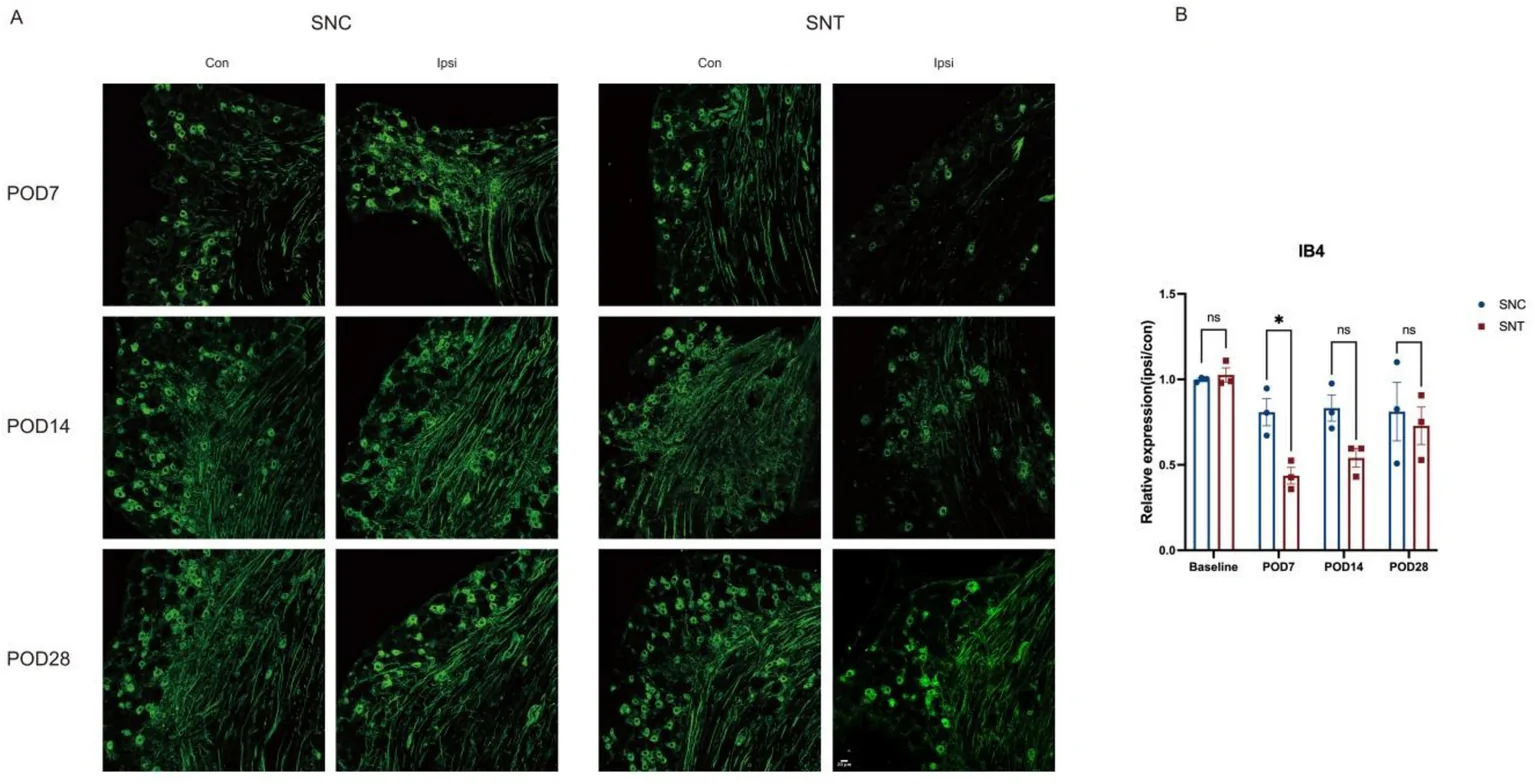
Isolectin B4 (IB4)-positive neuronal profiles in DRG following SNC and SNT procedures. **(A)** Representative immunofluorescence images showing IB4 (green) in contralateral (Con) and ipsilateral (Ipsi) L4-L6 DRG at POD7, 14, and 28. DAPI (blue). Scale bar = 20 μm. **(B)** Quantification of IB4 fluorescence intensity, expressed as the ipsilateral/contralateral ratio (primary metric, by design; see Section 2.6); IB4-positive profiles per section shown as a secondary measure. Mean ± SD. n = 3 mice per group per time point. SNC vs. SNT at POD7 was significantly higher for SNC after correction for multiple comparisons (*p = 0.0325; Supplementary Table S3); the within-SNT ipsilateral-versus-contralateral reduction reported in Section 3.3 is a separate comparison from this SNC-versus-SNT group comparison. See Supplementary Figure S2 (Nissl/TUBB3) for total-neuron context.

**Figure 6:**
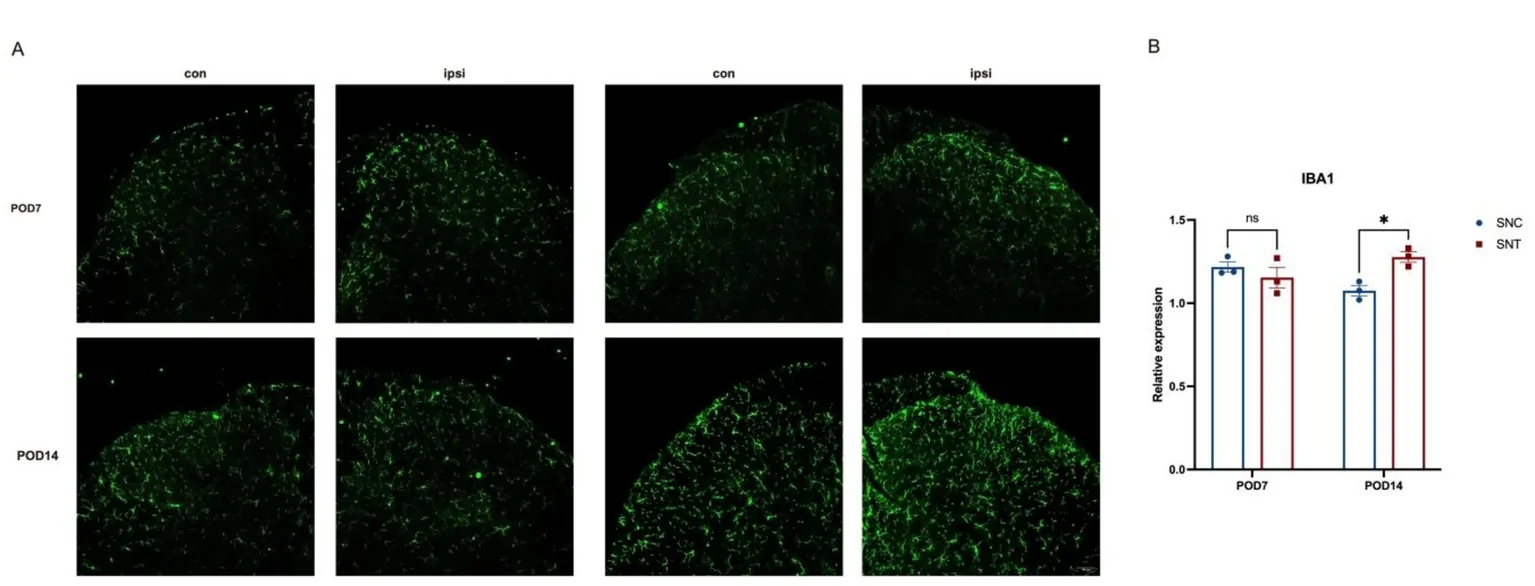
Microglial activation in the spinal dorsal horn. **(A)** Representative immunofluorescence images showing Iba1 (green) in contralateral (Con) and ipsilateral (Ipsi) spinal dorsal horn at POD7 and POD14 in SNC and SNT. Scale bar = 100 μm. **(B)** Quantification of Iba1 area fraction (primary metric); ipsi/contra ratio shown as secondary measure. Mean ± SD. *n* = 3 mice per group per time point. SNC vs. SNT at POD14 was statistically significant after correction for multiple comparisons (**p = 0.0173; Supplementary Table S3); see Section 3.5 and Supplementary Figure S3 for corroborating morphology-level (Cx3cr1/CD11b) evidence.

Sample size was determined *a priori* using power analysis based on preliminary ATF3-positive cell-count data from DRG sections (effect size = 0.8, alpha = 0.05, power = 0.8), yielding a minimum of 5 mice per group per time point for the primary wild-type behavioral endpoints. Histological endpoints in the reporter lines (AdvCre; Ai14, and MYCN-eGFP) used smaller, exploratory cohorts (*n* = 3/group/time point; Supplementary Table S1) that were not independently powered; we flag this explicitly as a limitation in the Discussion (Section 4) for those specific endpoints. Mice were assigned to Sham, SNC, and SNT groups using a computer-generated random-number sequence before surgery. Cage cards were coded by an investigator not involved in behavioral scoring or image quantification, and outcome assessors remained blinded to group allocation until the analysis dataset was finalized.

### 2.2 Sciatic nerve transection model

Animals were initially anesthetized with 2.5% isoflurane (RWD Life Science, China, catalog #R510-22-10) for induction and then maintained under 2% isoflurane anesthesia with an oxygen flow rate of 0.3–0.5 L/min. Under aseptic conditions, a skin incision was made on the lateral surface of the mouse’s thigh, and blunt dissection of the biceps femoris muscle was performed to expose the sciatic nerve by carefully separating it from the surrounding tissue. For the sciatic nerve transection procedure, the main trunk of the sciatic nerve was transected sharply at the mid-thigh level using microscissors (Fine Science Tools, USA, catalog #14094–11), and a 2–4 mm segment of the distal nerve stump was resected to prevent spontaneous reinnervation. The proximal stump was gently placed back into its original position. The muscle layer was closed with 5–0 Vicryl sutures (Ethicon, USA, catalog #J813G), and the skin was closed with wound clips. In the sham-operated mice, the sciatic nerve was exposed, but no further intervention was carried out.

### 2.3 Sciatic nerve crush model

For the sciatic nerve crush procedure, following the same surgical exposure described above, the sciatic nerve was gently freed from the surrounding connective tissue using fine forceps. Fine hemostatic forceps (Fine Science Tools, USA, catalog #13020–12) with smooth jaws were applied perpendicular to the nerve at the mid-thigh level. The nerve was crushed twice at the same site to ensure complete axon disruption: first for 15 s, followed by a 10-s interval, then a second crush for 15 s. Following the crush, the nerve became translucent at the injury site, confirming complete axonal interruption while preserving the epineurial sheath (An et al., 2022). After the surgery, the skin over the wound was sutured. In the sham-operated mice, the sciatic nerve was exposed, but no further intervention was carried out.

### 2.4 Behavioral tests

All behavioral tests were conducted between 9:00 a.m. and 5:00 p.m. by an experimenter blinded to the surgical procedures. Mice were acclimated to the testing apparatus for three consecutive days before baseline measurements.

Sensory function was assessed using the pinprick test as previously described (Painter et al., 2014). Mice were placed on a wire mesh platform and habituated for three sessions during the week before surgery. On each postoperative testing day, after a 30-min habituation period, the animal was gently restrained, and the plantar surface of the hind paw was stimulated with a 2 g von Frey filament at an intensity sufficient to indent but not puncture the skin. The lateral part of the plantar surface, corresponding to the sensory field of the sciatic nerve, was conceptually divided into five areas (from the most lateral toe to the heel), and the pinprick was applied twice to each area. A response was considered positive if the animal briskly withdrew its paw; each area was scored 1 if the animal responded to either application and 0 if it failed to respond to both, giving a summed score of 0–5 per paw (baseline scores of 4–5, reflecting intact sensory function across all five zones; Figure 7A). All scoring was performed blinded to the genotype and surgical group.

**Figure 7:**
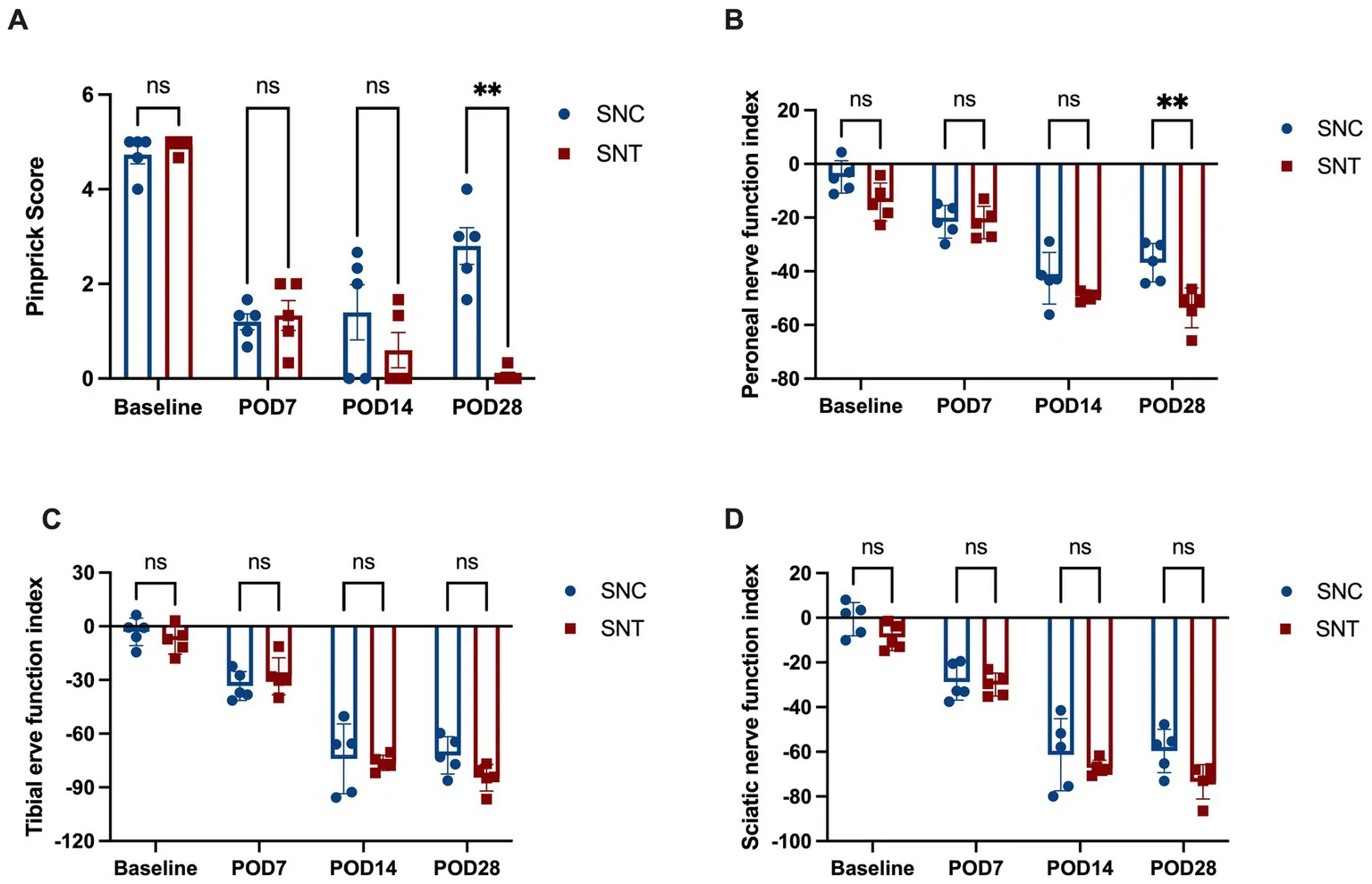
Comparison of sensory and motor functional restitution in SNC and SNT groups. **(A)** Pinprick response score at postoperative days 7, 14, and 28. **(B)** Peroneal functional index (PFI). **(C)** Tibial functional index (TFI). **(D)** Sciatic functional index (SFI). Data are mean ± SD. n = 5 mice per group per time point (see Supplementary Table S1). SNC vs. SNT at POD28 (corrected; Supplementary Table S3): sensory score, **p = 0.0073; PFI, **p = 0.0015; TFI, not significant (*p* = 0.238); SFI, not significant (*p* = 0.070). Motor recovery after SNC is therefore partial and measure-specific (evident in PFI) rather than uniform across all locomotor indices; see Section 3.2 and Section 4 for interpretation.

Motor function was assessed using the walking track analysis as previously described (Inserra et al., 1998). Mice were trained to walk along a 50 cm x 10 cm corridor lined with white paper. The hind paws were dipped in non-toxic black ink, and footprints were recorded. Three to five clear footprints per animal were used for analysis; footprints with smearing, overlap, or incomplete toe/pad contact were excluded. The following parameters were measured: print length (PL), toe spread (TS, distance between first and fifth toes), and intermediate toe spread (ITS, distance between second and fourth toes). Normal/maximum reference values (PLnorm, TSnorm, ITSnorm, TToeDispmax, and FToeDispmax) were obtained from the contralateral limb of the same animals. The sciatic functional index (SFI), tibial functional index (TFI), and peroneal functional index (PFI) were calculated as follows. Consistent with the experimental-minus-normal sign convention established by Bain et al. (1989), and adapted for the mouse by Inserra et al. (1998):


$$\begin{array}{l} \mathrm{SFI}=-38.3\;x\;(\mathrm{PL}-\text{PLnorm})/\text{PLnorm}+109.5\;x\;(\mathrm{TS}-\text{TSnorm})/\\ \text{TSnorm}+13.3\;x\;(\mathrm{ITS}-\text{ITSnorm})/\text{ITSnorm}-8.8. \end{array}$$

$$\begin{array}{l} \mathrm{TFI}=-37.2\;x\;(\mathrm{PL}-\text{PLnorm})/\text{PLnorm}+104.4\;x\;(\mathrm{TS}-\text{TSnorm})/\\ \text{TSnorm}+45.6\;x\;(\mathrm{ITS}-\text{ITSnorm})/\text{ITSnorm}-8.8. \end{array}$$

$$\begin{array}{l} \mathrm{PFI}=174.9\;x\;(\mathrm{PL}-\text{PLnorm})/\text{PLnorm}+80.3\;x\;\\ (\mathrm{TS}-\text{TSnorm})/\text{TSnorm}-13.4. \end{array}$$

For the behavioral dataset presented in Figure 7, wild-type mice were allocated to Sham (*n* = 6), SNC (*n* = 8), and SNT (*n* = 8) per time point. Exclusion criteria (inconsistent footprint quality precluding SFI/TFI/PFI calculation, or inconsistent pinprick responses across trials) were defined before data analysis began, and the resulting exclusion decisions were made by an assessor blinded to treatment group; this yielded a matched subset of *n* = 5 mice per group per time point for the figure and formal analysis, so that a balanced, quality-matched dataset could be compared across groups. Tissue for immunofluorescence in the AdvCre; Ai14 and MYCN-eGFP reporter lines was collected separately (*n* = 3 mice per genotype per group per time point; Supplementary Table S1); as noted in Section 2.1, these reporter-line histological cohorts were exploratory and not independently powered.

### 2.5 Tissue processing and immunohistochemistry

At 7, 14, and 28 days post-injury, mice were deeply anesthetized with 2% isoflurane and transcardially perfused with 4 °C phosphate-buffered saline (PBS) followed by 4% paraformaldehyde (PFA) in PBS. The L4-L6 spinal cord segments, ipsilateral and contralateral L4-L6 DRG, and sciatic nerve segments (including the injury site) were carefully dissected and post-fixed in 4% PFA for 4–6 h at 4 °C. Tissues were then cryoprotected in 30% sucrose in PBS for 72 h at 4 °C, embedded in OCT compound (Sakura Finetek, Japan), and frozen on dry ice. Using a cryostat (CM3050S, Leica, Germany), DRG and sciatic nerve tissues were sectioned longitudinally at 10 μm thickness, while spinal cord tissues were sectioned coronally at 20 μm thickness. Sections were collected on Superfrost Plus slides (Thermo Fisher Scientific, USA) and stored at −80 °C until use. For immunohistochemistry, sections were thawed, washed in PBS (Servicebio, China, catalog #G0002), and blocked with 5% normal donkey serum (Jackson ImmunoResearch, USA, catalog #017–000-121) in PBS containing 0.3% Triton X-100 (Sigma-Aldrich, catalog #T8787) for 60 min at room temperature. Sections were then incubated overnight at 4 °C with primary antibodies diluted in blocking solution. The following primary antibodies or labeling reagents were used: myelin basic protein/myelin marker (mouse monoclonal, 1:200, Santa Cruz Biotechnology, catalog #sc-69894, RRID: AB_1122797), ATF3 (rabbit polyclonal, 1:500, Santa Cruz Biotechnology, catalog #sc-188, RRID: AB_2258513), IB4 conjugated to Alexa Fluor 488 (1:200, Thermo Fisher Scientific, catalog #I21411, RRID: AB_2571615), Iba1 (mouse monoclonal, 1:500, Santa Cruz Biotechnology, catalog #sc-32725, RRID: AB_627547), NeuN (mouse monoclonal, 1:200, Millipore, catalog #MAB377, RRID: AB_2298772), NeuroTrace 500/525 green fluorescent Nissl stain (1:200, Thermo Fisher Scientific, catalog #N21480, RRID: AB_2620000), TUBB3 (mouse monoclonal, 1:500, BioLegend, catalog #801213, RRID: AB_2313773), Cx3cr1 (rabbit polyclonal, 1:200, Santa Cruz Biotechnology, catalog #sc-30030, RRID: AB_631354), and CD11b (rat monoclonal, 1:200, BioLegend, catalog #101202, RRID: AB_312789), and endogenous reporter fluorescence from AdvCre; Ai14 tdTomato and MYCN-eGFP mice. Primary-antibody specificity was assessed using a combination of vendor validation data, Western blot validation using knockout tissue lysates where available (ATF3−/− tissue for ATF3 and Iba1−/− tissue for Iba1), expected cellular and subcellular staining patterns, and comparison with published reports. Omission of the primary antibody was used only to assess nonspecific signal from secondary antibodies and was not considered sufficient evidence for primary-antibody specificity. After primary incubation, sections were washed in PBS and incubated with appropriate secondary antibodies (Alexa Fluor 488 donkey anti-mouse IgG, Thermo Fisher Scientific, catalog #A-21202, RRID: AB_141607; Alexa Fluor 555 donkey anti-rabbit IgG, Thermo Fisher Scientific, catalog #A-31572, RRID: AB_162543; Alexa Fluor 647 donkey anti-rabbit IgG, Thermo Fisher Scientific, catalog #A-31573, RRID: AB_2536183; and Alexa Fluor 594 donkey anti-rat IgG for CD11b detection, Thermo Fisher Scientific, catalog #A-21209, RRID: AB_2535795; 1:500) for 1 h at room temperature. Sections were then washed and counterstained/mounted with Fluoromount-G with DAPI (SouthernBiotech, USA, catalog #0100–20). Negative controls omitting primary antibody were included in each staining run.

### 2.6 Image acquisition and analysis

Fluorescence images were acquired using an Olympus FV3000 confocal microscope with FV31S-SW software and saved as .oir files. Identical acquisition settings (laser power, PMT gain, PMT offset, pinhole at 1 Airy unit, scan speed, resolution, zoom) were used for all sections. To render the background of a specific channel black for display without altering raw data, the black point slider in the Brightness/Contrast panel of FV31S-SW was adjusted rightward for that channel. Alternatively, after importing .oir files into Fiji/ImageJ via the Bio-Formats plugin, the same display-only adjustment was achieved by moving the Minimum slider in the Brightness/Contrast panel (Ctrl+Shift+I). Original raw data remained unchanged in all cases. For each animal, 3–5 non-overlapping sections per region (ipsilateral and contralateral L4-L6 DRG, spinal cord ventral horn, and dorsal horn) were imaged using an Olympus FV3000 confocal microscope with identical settings. For each section, 2–4 fields of view were captured from predefined anatomical regions; per-animal values were obtained by averaging across sections and fields before entry into group-level statistics, such that the animal, not the section or field of view, is the statistical unit for all histological quantification. ATF3-positive cells were counted using Fiji/ImageJ (Cell Counter plugin) in a blinded manner and are reported as the percentage of ATF3-positive cells relative to total DAPI-positive (or NeuN-positive, where co-stained) profiles per section; this percentage, not an ipsilateral/contralateral ratio, is the metric reported for ATF3 throughout the manuscript (Figure 4B). MYCN-eGFP was quantified as mean fluorescence intensity (mean-gray-value measurement, Fiji/ImageJ, with the analyst blinded to group allocation) and is reported primarily as an absolute value per section (arbitrary units); the ipsilateral/contralateral ratio is reported only as a secondary, descriptive measure for MYCN-eGFP. For Iba1, the area fraction of Iba1-positive signal was measured after thresholding using the Otsu method and is reported as the primary metric (Figure 6B); an ipsilateral/contralateral ratio is shown only as a secondary, descriptive measure. For IB4, the mean intensity of IB4 fluorescence was measured and is reported as the ipsi/con ratio; because IB4 signal reflects both the number of labeled profiles and per-cell binding intensity, we used this within-animal, paired ratio (rather than an absolute value) to provide a more objective, inter-animal-staining-variability-controlled readout of injury-induced change. Data are presented as mean ± SD for normally distributed data, or median [IQR] for non-normally distributed data (see Supplementary Table S3 for assumption-test results).

### 2.7 Statistical analysis

All data were analyzed using GraphPad Prism 9.0. Normality was assessed for each endpoint and group/time point using the Shapiro–Wilk test; homogeneity of variances was assessed using Levene’s test or Brown-Forsythe test, as appropriate, to determine whether parametric or non-parametric tests were used for each endpoint (see below); exact normality/variance test statistics are available from the authors upon request. For normally distributed data with equal variances, parametric tests were used (two-way repeated-measures ANOVA, one-way ANOVA, or two-way ANOVA followed by Tukey’s or Sidak’s *post hoc* test, as specified in each figure legend). For longitudinal data, sphericity was assessed using Mauchly’s test; if violated, the Greenhouse–Geisser correction was applied. For non-normally distributed data or data with unequal variances, Kruskal-Wallis test with Dunn’s *post hoc* test or other appropriate nonparametric tests were used, and data were summarized as median [IQR]. Statistical results are reported in APA format, including test statistic, degrees of freedom, and exact *p* values (for example, F(2, 15) = 6.42, *p* = 0.010). Exact *p* values are reported for *p* > 0.001, and *p* < 0.001 is used only for smaller values. Sex was included as a factor in initial multi-way ANOVA; no sex interaction was detected in the present dataset (*p* > 0.05), and data from male and female mice were therefore pooled. The MYCN-eGFP quantification in Figure 1B was analyzed by two-way ANOVA (Group x Time) with Sidak-corrected multiple comparisons; the specific *post hoc* test used for each other endpoint is stated in the corresponding figure legend, and full test statistics for all endpoints are compiled in Supplementary Table S3.

## 3 Results

### 3.1 Histological evidence of axonal degeneration and regeneration after SNC

To visualize sensory axonal labeling and assess axonal degeneration and regeneration dynamics, we utilized AdvCre; Ai14 mice, in which Advillin-positive peripheral sensory neurons are constitutively labeled with tdTomato. We performed multi-channel immunofluorescence staining on the sciatic nerve after SNC injury to verify this dynamic process at the histological level: the green channel labeled myelin to demonstrate the structural integrity of the nerve bundle, the red channel labeled tdTomato to trace the distribution of sensory neuron axons, and the merged channel showed the spatial localization relationship between the two (Figure 3). At post-operation day 7 (POD7), the nerve in the early stage of injury presented typical pathological features after injury: the myelin fibers of the proximal nerve still maintained a regular bundle arrangement, with rich and continuous tdTomato signal, and the two were well co-localized, suggesting that the proximal nerve structure was not significantly affected. In the crush injury site, the myelin signal showed obvious dispersion and disorder, and the normal bundle structure completely disappeared, indicating that the injury induced acute myelin disintegration. At the same time, the tdTomato signal in this area was significantly reduced, with only a small amount of fragmented axon residues left, and a large number of punctate tdTomato-associated signals, consistent with fragmented axonal labeling during Wallerian degeneration, were visible in the merged channel. In the distal nerve of the injury site, the tdTomato signal almost completely disappeared, with only a small amount of disordered myelin residue signal left, directly confirming that after axon fracture, the distal segment underwent Wallerian degeneration.

### 3.2 Partial functional recovery after SNC versus persistent deficit after SNT

To correlate our morphological and molecular findings with functional outcomes, we assessed sensory and motor recovery longitudinally. The SNC group demonstrated a gradual and significant restitution of sensory function, reaching approximately 60% of baseline by POD28 (Figure 7A; *p* < 0.05 vs. POD7). Conversely, the SNT group showed a complete and persistent loss of sensory function, with no signs of neurological restoration throughout the 28-day observation period (Figure 7A; *p* < 0.01 vs. SNC at POD28). Motor function was assessed by the peroneal (PFI), tibial (TFI), and sciatic (SFI) functional indices (Figures 7B–D); both groups exhibited an initial severe decline at POD7. By POD28, the SNC group showed a significant within-group improvement in PFI relative to POD14 (Figure 7B; *p* < 0.05 vs. POD14), and PFI was significantly better in SNC than SNT at POD28 (*p* = 0.0015, corrected; Supplementary Table S3). TFI and SFI showed a similar numerical trend toward better SNC recovery but did not reach statistical significance for the SNC-versus-SNT comparison at POD28 (TFI, *p* = 0.238; SFI, *p* = 0.070; Supplementary Table S3). We therefore describe SNC motor recovery as partial and index-specific—clearest for PFI—rather than as uniform restitution across all three locomotor indices, while SNT motor function remained at or near its post-injury minimum throughout the observation period. The raw footprint measurements are listed in Supplementary Table S2. These data indicate that the preserved structural scaffold in the SNC model is associated with meaningful functional recovery, supporting its use for studying endogenous repair mechanisms; we present this functional dataset primarily as a framing/validation readout for the cellular findings that follow, rather than as the central novel claim of this study (see Section 4).

### 3.3 Injury modality dictates neuronal subtype-specific responses in the DRG

We next investigated the response of sensory neuron cell bodies in the DRG to the two injury types. ATF3, a canonical marker of neuronal injury and regeneration initiation, was virtually absent in contralateral (uninjured) DRG neurons. In the ipsilateral DRG, both models induced a peak in ATF3 expression at POD7 (Figures 4A,B). However, the temporal dynamics diverged sharply thereafter. In the SNC group, ATF3 expression rapidly declined, returning to near-baseline levels by POD28 (Figure 4B). This transient expression pattern is consistent with a self-limited regenerative program. In the SNT group, ATF3 expression remained elevated relative to SNC at POD14 and POD28, reaching statistical significance at POD28 (**p = 0.0014, corrected; Supplementary Table S3), consistent with a state of chronic neuronal stress and failed target reconnection (Figure 4B); we have not separately tested the POD14 SNC-versus-SNT comparison and describe that time point qualitatively. We then examined the fate of IB4-positive non-peptidergic nociceptors, a key subpopulation of small-diameter DRG neurons critically dependent on peripheral target-derived trophic support. SNT induced a marked and rapid reduction in IB4-positive neuronal profiles, to approximately 50% of baseline by POD7 (*p* < 0.05 vs. contralateral), which only partially recovered to 60% by POD28 (Figures 5A,B). In striking contrast, the SNC group showed only a minimal, non-significant reduction at any time point (Figure 5B). To help interpret this reduction, we performed co-staining for the pan-neuronal markers Nissl substance and TUBB3 in ipsilateral versus contralateral DRG at POD7 after SNT (Supplementary Figure S2); the overall Nissl-positive/TUBB3-positive neuronal population appeared grossly preserved in density and morphology on the ipsilateral side, arguing against wholesale neuronal loss at this time point and supporting the interpretation that the reduction in IB4 labeling may, at least in part, reflect a phenotypic or lectin-binding change rather than neuronal death (see Discussion); we did not perform TUNEL or cleaved caspase-3 staining, and this remains an important direction for directly distinguishing these possibilities. Co-staining for ATF3 and IB4 revealed that these two markers labeled largely non-overlapping neuronal populations (Figure 8), with ATF3 predominantly in larger-diameter neurons and IB4 in smaller ones. This spatial segregation suggests that different neuronal subtypes exhibit distinct intrinsic sensitivities and injury responses. The pronounced and sustained reduction of IB4-positive labeling after SNT highlights a differential response of the IB4-positive phenotype in this nociceptor population to complete axotomy and prolonged neurotrophic factor deprivation; as discussed below, this reflects reduced IB4 labeling and does not, by itself, establish selective loss of IB4-lineage neurons. This finding has implications for post-traumatic sensory dysfunction.

**Figure 8:**
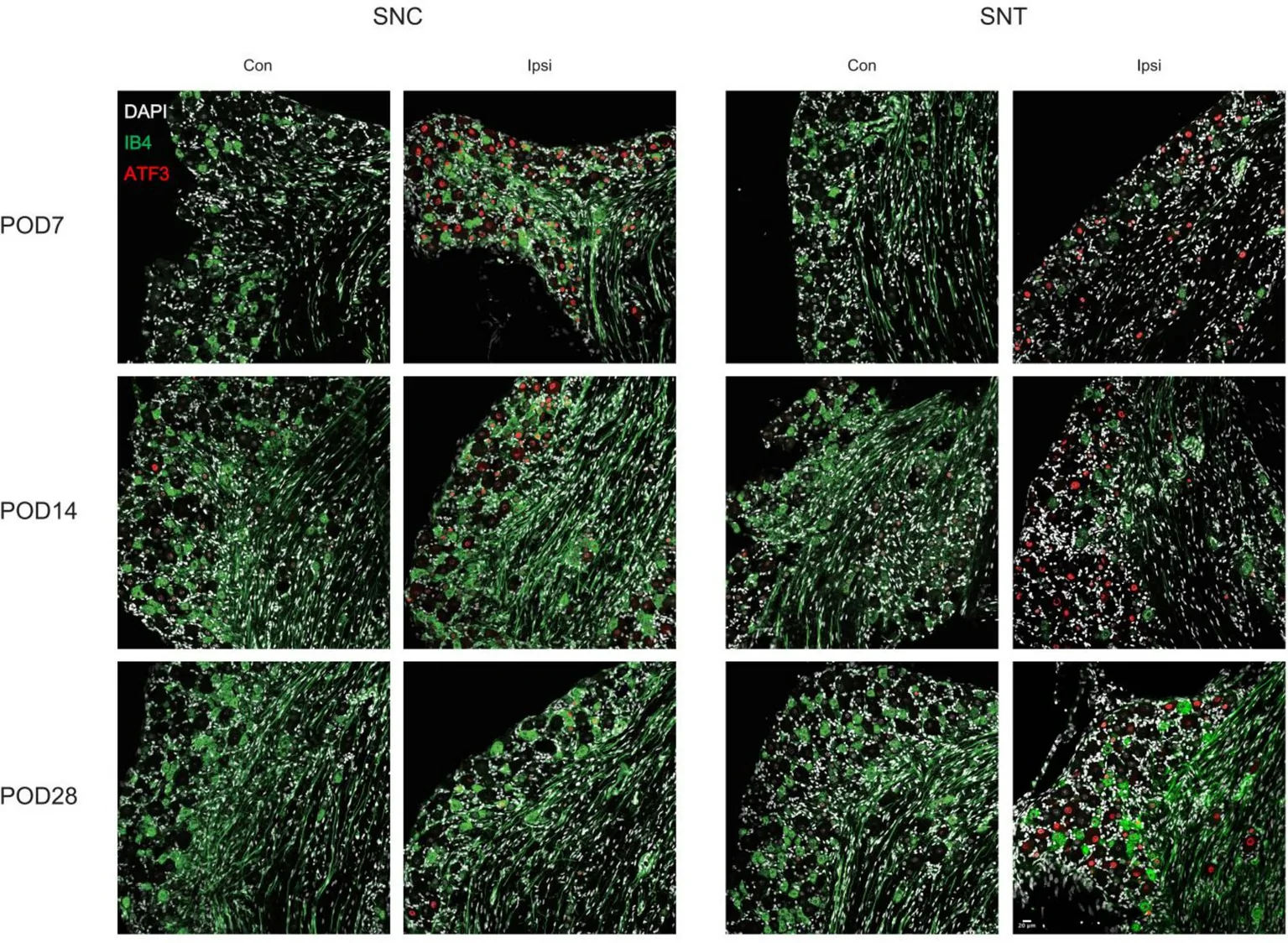
Spatially distinct expression patterns of ATF3 and IB4 in DRG neurons. Representative merged immunofluorescence images showing co-localization of ATF3 (red) and IB4 (green) in the contralateral (Con) and ipsilateral (Ipsi) sides of SNC and SNT groups at POD7, POD14, and POD28. Note the largely non-overlapping populations, with ATF3 predominantly in larger neurons and IB4 in smaller-diameter neurons. Scale bar = 20 μm.

### 3.4 Peripheral nerve injury induces bilateral MYCN-eGFP responses in spinal ventral-horn neurons

To examine MYCN expression and its association with neuronal injury responses after peripheral nerve injury, MYCN-eGFP reporter mice were subjected to Sham surgery, SNC, or SNT, and spinal ventral horn sections were analyzed at POD7 and POD14 by immunofluorescence for MYCN-eGFP, the injury-induced transcription factor ATF3, and DAPI nuclear counterstaining (Figure 1A). To rigorously quantify this response, we measured MYCN-eGFP mean fluorescence intensity per section (blinded quantification, Fiji/ImageJ) in all six Group x Side combinations (Sham/SNC/SNT x ipsilateral/contralateral) at both time points (*n* = 3 animals/group/time point, the MYCN-eGFP reporter cohort defined in Section 2.1 and Supplementary Table S1) and analyzed the data by two-way ANOVA with Sidak-corrected multiple comparisons (Figure 1B; full statistics in Supplementary Table S3).

Consistent with its established role as a canonical neuronal injury marker, ATF3 was exclusively detected in ipsilateral spinal ventral-horn neurons in both SNC and SNT models. ATF3 expression was prominent at POD7 and largely diminished by POD14, displaying a typical injury-induced transient expression pattern.

MYCN-eGFP intensity was significantly elevated above the sham baseline in every injury/side combination at POD7: SNC-ipsilateral (255.7 ± 10.3 AU) and SNC-contralateral (122.7 ± 23.2 AU) versus Sham-contralateral (2.4 ± 1.2 AU), t(4) = 24.52, *p* = 0.0002 and t(4) = 11.59, *p* = 0.0047, respectively; SNT-ipsilateral (80.2 ± 7.9 AU) and SNT-contralateral (62.2 ± 16.8 AU) versus Sham-contralateral, t(4) = 9.85, *p* = 0.0089 and t(4) = 7.99, *p* = 0.0198, respectively (equivalent comparisons versus Sham-ipsilateral were also significant). This confirms that MYCN induction is bilateral and occurs in both injury models rather than being restricted to SNT.

The acute (POD7) MYCN response was numerically and statistically greatest after SNC: SNC-ipsilateral significantly exceeded SNT-ipsilateral (t(4) = 13.07, *p* = 0.0030) and SNT-contralateral (t(4) = 14.46, *p* = 0.0020), and also exceeded contralateral SNC itself (t(4) = 9.04, *p* = 0.0124, reflecting a within-group ipsilateral-greater-than-contralateral gradient in SNC). SNC-contralateral versus SNT-contralateral did not differ significantly at POD7 (*p* = 0.30).

By POD14, MYCN intensity had declined significantly from its POD7 peak in every injured group (Sham groups showed no change over time, both *p* > 0.4), but the magnitude of decline differed by injury modality: SNC-ipsilateral fell by 231.1 AU (90% of peak; t(4) = 21.28, *p* < 0.0001) and SNC-contralateral by 112.5 AU (92% of peak; t(4) = 9.998, *p* = 0.0006), whereas SNT-ipsilateral fell by 48.5 AU (60% of peak; t(4) = 5.63, *p* = 0.0049) and SNT-contralateral by 36.1 AU (58% of peak; t(4) = 3.98, *p* = 0.0164). All four injured groups nonetheless remained significantly above sham at POD14 (*p* < = 0.016) with the exception of SNC-contralateral versus SNT-contralateral, which converged (*p* = 0.088), and most SNC-versus-SNT comparisons at POD14 were not statistically significant.

MYCN-eGFP signals extensively co-localized with ATF3-positive neurons in the ipsilateral injured spinal cord (yellow arrowheads, Figure 1A), indicating that MYCN is induced in acutely injured, ATF3-expressing ventral-horn neurons. However, the presence of strong MYCN signals in contralateral, uninjured neurons and in ipsilateral non-ATF3-expressing neurons indicates that MYCN induction is not restricted to directly injured neurons and is governed by additional mechanisms beyond the canonical ATF3-marked injury response. To help confirm the identity of MYCN-eGFP-positive cells, we performed co-staining with NeuN in ipsilateral ventral horn at POD14 in SNC and SNT (Supplementary Figure S1); MYCN-eGFP-positive cells co-localized with large, multipolar NeuN-positive somata consistent with motor neuron morphology and laminar position, though we note that NeuN is a pan-neuronal marker, not a motor-neuron-specific one, and does not distinguish motor neuron subtypes as definitively as ChAT, Islet1/2, or Hb9, which remain a planned confirmatory follow-up, given the known diversity of spinal motor neuron subtypes (Liau et al., 2021). Pending that confirmation, we describe these cells throughout the manuscript as spinal ventral-horn neurons with morphology, laminar position, and NeuN co-labeling consistent with motor neurons, rather than as definitively identified motor neurons. We also emphasize that our MYCN conclusions are based on MYCN-eGFP reporter fluorescence; we have not independently confirmed endogenous MYCN protein or transcript expression by immunostaining, RNAscope, or qPCR, and we therefore distinguish MYCN-eGFP reporter activity from confirmed endogenous MYCN expression throughout the manuscript.

Taken together, these results indicate that MYCN is an injury-general, bilaterally-induced cellular response of spinal ventral-horn neurons rather than an SNT-exclusive phenomenon, whose acute (POD7) amplitude is greatest after the regeneration-permissive SNC injury, while its relative persistence to POD14 is greater after the persistent-disconnection SNT injury. We hypothesize that the bilateral component of this response reflects a contralateral (“mirror”) plasticity program (Won and Lee, 2015; Drinovac Vlah and Bach-Rojecky, 2024); a plausible but untested circuit substrate is trans-segmental signaling via commissural interneurons, which we present explicitly as a hypothesis, since no interneuron-specific manipulation or tracing was performed in this study.

### 3.5 Microglial activation in the spinal dorsal horn after SNC and SNT

Finally, we assessed the central neuroinflammatory response by examining Iba1, a marker of microglial activation, in the spinal dorsal horn. Both SNC and SNT induced microglial activation on the ipsilateral side at POD7 and 14 (Figure 6A). The SNT group showed a numerically larger Iba1 area fraction than SNC at POD14, and this between-group difference was statistically significant after correction for multiple comparisons (**p = 0.0173; Supplementary Table S3; Figure 6B), consistent with a more protracted microglial response after SNT. To characterize this response beyond area-fraction quantification, we performed co-staining for Cx3cr1 and CD11b in the ipsilateral versus contralateral dorsal horn at POD7 after SNT (Supplementary Figure S3); ipsilateral microglia showed a denser field of Cx3cr1-positive/CD11b-positive cells with a less ramified, more amoeboid morphology relative to the contralateral side, qualitatively consistent with a shift toward an activated phenotype.

## 4 Discussion

This study provides a comparative analysis of the cellular responses to sciatic nerve crush versus transection. Our findings move beyond simply confirming that crush injuries heal better; instead, we delineate differences in the cellular responses, and in spinal cord signatures, that distinguish these two injury modalities. We show that the SNC model is characterized by a transient, self-limited response that accompanies successful regeneration, while the SNT model is associated with persistent neuronal stress, a differential subtype-specific response in the DRG, and a bilateral spinal MYCN response whose temporal persistence, but not the presence of the response, differs between the two injury modalities, within a dorsal-horn microglial response.

Our tdTomato tracing data in the AdvCre; Ai14 mice provide a dynamic view of sensory axonal labeling during degeneration and regeneration. The rapid restoration of distal tdTomato signal in the SNC model by 28 days illustrates how the preserved endoneurial tubes act as a guide for regenerating axons (George et al., 2004; He et al., 2023). The transient proximal accumulation of tdTomato may reflect altered distribution of sensory axonal labeling during the early injury response. The permanent loss of signal in the distal stump after SNT underscores the consequence of losing this physical guidance structure, leaving the disconnected axons to undergo Wallerian degeneration without the possibility of target re-innervation (Chen et al., 2025). This contrast supports the SNC model as a useful platform for studying the intrinsic cellular machinery of successful nerve repair.

The DRG analysis reveals a nuance in the neuronal response to injury. The transient ATF3 expression in SNC aligns with its role as an early, pro-regenerative transcription factor (Gey et al., 2016). Its persistent elevation in SNT, however, suggests a shift from a regenerative signal toward a marker of chronic cellular stress. More importantly, the pronounced and sustained reduction of IB4 labeling after SNT (Figure 5) identifies the IB4-positive phenotype in this subpopulation as particularly responsive to the consequences of complete axotomy and prolonged target deprivation (Price and Flores, 2007); we use this phrasing to describe a change in marker labeling, not a confirmed loss of these neurons. We interpret this cautiously: reduced IB4 binding after axotomy has been shown in other systems to reflect a phenotypic or lectin-binding switch rather than necessarily indicating neuronal death — for example, IB4-binding DRG neurons are known to shift dependence from NGF- to GDNF-family signaling during development and after injury (Molliver et al., 1997), and pericellular IB4-binding ring structures reorganize substantially in the DRG after peripheral nerve injury without a proportional loss of total neuron counts (Li and Zhou, 2001). Our new Nissl/TUBB3 co-staining (Supplementary Figure S2) similarly shows a grossly preserved total DRG neuronal population on the injured side at POD7, consistent with a phenotype-switch component to the observed reduction in IB4 labeling, though we cannot exclude a contribution from neuronal loss without TUNEL or cleaved caspase-3 data. This differential marker response, whatever its precise cellular basis, has implications for understanding post-traumatic sensory dysfunction, as altered signaling from this neuronal subset can contribute to aberrant processing and central sensitization; because pain-like behavior was assessed here only with the pinprick test, we limit our claims to sensory dysfunction rather than neuropathic pain specifically, and note that von Frey, cold-allodynia, or thermal-hypersensitivity assays would be required to directly support pain-specific conclusions. This differential marker response supports considering neuronal subtype in studies of nerve injury and pain, rather than treating the DRG as a homogeneous population (Usoskin et al., 2015).

Consistent with its well-established role as a canonical marker for acutely injured neurons, ATF3 expression was strictly confined to ipsilateral spinal ventral-horn neurons following both SNC and SNT, robustly induced at POD7, and nearly abolished by POD14. This transient and injury-side-specific expression pattern validates the specificity of our injury models and indicates that only directly lesioned ventral-horn neurons engage the classical ATF3-dependent injury transcriptional program (Seijffers et al., 2007).

In contrast, MYCN-eGFP exhibited a non-canonical expression profile following peripheral nerve injury. Our quantitative analysis (Figure 1B) shows that MYCN was induced bilaterally at POD7 in both SNC and SNT, refuting an SNT-exclusive interpretation; the acute amplitude of this response was in fact greatest after SNC. This bilateral, injury-general induction pattern is consistent with a growing literature describing contralateral (“mirror”) spinal cord changes after unilateral peripheral nerve injury, including contralateral metabolic activation in the spinal ventral horn (Won and Lee, 2015) and broader contralateral structural and molecular changes described across pain and injury models (Drinovac Vlah and Bach-Rojecky, 2024). We propose that contralateral MYCN induction may reflect interneuronal communication, trans-segmental signaling via spinal interneuron circuits (Sengupta and Bagnall, 2023), or homeostatic plasticity rather than direct axotomy; we state this explicitly as a hypothesis, since no interneuron-specific manipulation, tracing, or lesion of candidate commissural pathways was performed in this study.

MYCN intensity declined by more than 90% from POD7 to POD14 in SNC but by only approximately 60% in SNT, such that SNT shows relatively greater persistence of its own, lower-amplitude peak. These within-group POD7-versus-POD14 comparisons show that each injury type has its own temporal trajectory; we have not formally tested an injury x time (or injury x side x time) interaction term for MYCN-eGFP intensity. We therefore describe this cautiously as a numerical, descriptive difference in amplitude and temporal pattern between SNC and SNT, rather than as statistically confirmed evidence that the two injury modalities differ in the kinetics of the bilateral MYCN response, and we identify a formal interaction analysis as a specific priority for a future revision or follow-up study. Colocalization of MYCN-eGFP and ATF3 in ipsilateral ventral-horn neurons (Figure 1A) confirms that peripheral nerve injury is sufficient to trigger MYCN expression in directly damaged neurons, while the presence of MYCN in contralateral and non-ATF3 ipsilateral neurons indicates that additional, ATF3-independent mechanisms also contribute. Together, these findings identify MYCN-eGFP as an injury-associated cellular response that extends beyond classically injured neurons, highlighting a bilateral and dynamically regulated signal in the spinal cord after peripheral nerve injury. This bilateral MYCN response also means that the contralateral side cannot be assumed to be an entirely unaffected control: for ATF3, IB4, and Iba1 we used the contralateral side as an internal control because those specific markers are, to our knowledge, injury-restricted and not reported to change contralaterally, but we have not directly tested contralateral ATF3, IB4, or Iba1 against a sham comparator in this study, and we flag this as a limitation; sham comparisons for these endpoints, in addition to the sham-controlled MYCN-eGFP analysis already performed, would further strengthen this assumption in future work.

The differential microglial response in the spinal dorsal horn further distinguishes the two models. The microglial response after SNC may accompany debris clearance and other injury-associated processes during regeneration (Karam et al., 2025). In contrast, SNT showed a significantly larger Iba1 area fraction at POD14 (Figure 6B; **p = 0.0173, corrected), together with denser, less-ramified Cx3cr1-positive/CD11b-positive cells on the injured side at POD7 (Supplementary Figure S3); together these findings are consistent with, but do not statistically confirm, a more sustained state of central inflammation after SNT. Microglial activation is heterogeneous, and different injury contexts are associated with distinct phenotypic marker profiles beyond a single area-fraction measure (Li et al., 2013); our Cx3cr1/CD11b data provide morphology-level, but not mechanistic, support for this heterogeneity. Chronic neuroinflammation is a well-established driver of central sensitization in the broader pain literature (Inoue and Tsuda, 2018), and pro-inflammatory cytokines such as TNF and IL-1beta have been proposed to inhibit axonal growth cone progression (Garcia-Dominguez, 2025); we did not directly measure these cytokines, so this remains a literature-grounded hypothesis rather than a finding of the present study, and we describe the SNT dorsal horn as showing a more sustained microglial response rather than characterizing it in terms of permissiveness for regeneration. Establishing the chronicity and precise molecular phenotype of this microglial response—for example through POD28 quantification and additional activation markers such as CD68, p-p38, TMEM119, or P2RY12—remains an important direction for future work.

Our findings have two data-grounded implications for future work. First, SNC and SNT engage quantitatively distinct DRG programs (transient versus chronic ATF3; preserved versus reduced IB4-positive profiles), suggesting that peripheral-repair strategies might usefully be tailored to injury modality, for example through neurotrophic factor supplementation (e.g., GDNF-family ligands) aimed at supporting IB4-lineage neurons after complete axotomy. Second, the bilateral, divergent-kinetics MYCN response we describe is a candidate cellular readout of differential injury responses in the spinal cord, and a starting point—rather than a validated mechanism—for future studies using interneuron-specific tracing or manipulation to test the circuit basis of this contralateral signal.

## 5 Conclusion

In conclusion, this study characterizes divergent cellular responses to sciatic nerve crush versus transection. SNC is associated with a transient, self-limited DRG response and relatively preserved IB4-positive neuronal labeling, whereas SNT is associated with persistent DRG stress, a sustained reduction in IB4-positive neuronal profiles, and a more prolonged dorsal-horn microglial response. In the spinal cord, MYCN-eGFP signal is bilaterally increased in ventral-horn neurons in both SNC and SNT by POD7, with a greater acute amplitude after SNC and relatively greater persistence after SNT. These findings indicate that the two injury modalities produce overlapping but quantitatively and temporally distinct cellular responses. The bilateral MYCN-eGFP response may represent a contralateral (“mirror”) spinal response, although its functional significance and underlying circuitry remain to be determined. Overall, these findings support the use of SNC and unrepaired SNT as complementary models of regeneration-permissive and persistent-disconnection injuries, respectively, and identify bilateral MYCN-eGFP dynamics as a candidate cellular readout for future mechanistic investigation. Confirming ventral-horn neuronal identity with motor-neuron-specific markers (ChAT, Islet1/2), formally testing an injury x time interaction for the MYCN-eGFP response, and extending behavioral assessment to pain-specific measures (von Frey, thermal, cold) remain important priorities before therapeutic extrapolation.

## Data Availability

The original contributions presented in the study are included in the article/supplementary material, further inquiries can be directed to the corresponding authors.
