# Supplementary material for: Divergent cellular responses to sciatic nerve crush versus transection: bilateral MYCN induction and distinct DRG neuronal responses
Source: Front Cell Neurosci. 2026 Sep 28;20:1922426. doi: 10.3389/fncel.2026.1922426 (PMC13654848; doi:10.3389/fncel.2026.1922426)
Supplement: Supplementary file 1 [file Supplementary_file_1.docx]

# **Supplementary Figures**

**Supplementary Figure S1. MYCN-eGFP and NeuN co-staining in ipsilateral spinal ventral horn**

Representative confocal images; MYCN-eGFP-positive cells co-localize with large NeuN-positive somata consistent with motor neuron morphology. DAPI (gray), MYCN (green), NeuN (red).

**Supplementary Figure S2. Nissl and TUBB3 co-staining in DRG**

Representative confocal images showing a grossly preserved total neuronal population of L4 DRG on the injured side, POD7 after SNT. Contralateral (Con), ipsilateral (Ipsi). DAPI (gray), Nissl (green), TUBB3 (red).

**Supplementary Figure S3. Cx3cr1 and CD11b co-staining in spinal dorsal horn,**

Representative confocal images showing a denser field of less-ramified, more amoeboid-appearing microglia on the ipsilateral side of the dorsal horn, 7 days after SNT, providing morphology-level evidence of microglial activation beyond Iba1 area fraction. Contralateral (Con), ipsilateral (Ipsi). DAPI (gray), Cx3cr1 (green), CD11b (red).

**Supplementary Tables**

**Table S1. Animal allocation by genotype, injury group, time point, and assay**

| **Genotype** | **Group** | **POD7 — Behavior/Tissue** | **POD14 — Behavior/Tissue** | **POD28 — Behavior/Tissue** | **Group total** |
| --- | --- | --- | --- | --- | --- |
| Wild type | Sham | 6 | 6 | 6 | 18 |
| Wild type | SNC | 8 | 8 | 8 | 24 |
| Wild type | SNT | 8 | 8 | 8 | 24 |
| AdvCre;Ai14 | Sham | 3 | 3 | 3 | 9 |
| AdvCre;Ai14 | SNC | 3 | 3 | 3 | 9 |
| AdvCre;Ai14 | SNT | 3 | 3 | 3 | 9 |
| MYCN-eGFP | Sham | 3 | 3 | 3 | 9 |
| MYCN-eGFP | SNC | 3 | 3 | 3 | 9 |
| MYCN-eGFP | SNT | 3 | 3 | 3 | 9 |
| Total |  | 40 | 40 | 40 | 120 |

# **Table S2 Raw Footprint Measurements**

| **Group** | **Order** | **PL(mm)** | **TS(mm)** | **ITS(mm)** | **PLnorm (mm)** | **TSnorm (mm)** | **ITSnorm (mm)** | **SFI** | **TFI** | **PFI** |
| --- | --- | --- | --- | --- | --- | --- | --- | --- | --- | --- |
| SNC_Baseline | 1 | 21.8 | 40.6 | 17.6 | 21 | 40 | 20.3 | -10.1 | -14.4 | -5.4 |
| SNC_Baseline | 2 | 19.5 | 49.3 | 27.1 | 19.7 | 42.8 | 27.9 | 8 | 6.3 | -3.1 |
| SNC_Baseline | 3 | 21.4 | 38.3 | 22.8 | 21.3 | 37.6 | 22.4 | -6.4 | -5.9 | -11.2 |
| SNC_Baseline | 4 | 22.4 | 44.6 | 19.6 | 23 | 40 | 22 | 3.4 | -0.8 | -9 |
| SNC_Baseline | 5 | 20.4 | 46.7 | 25.3 | 19.5 | 41.6 | 27.2 | 2.1 | -0.7 | 4.4 |
| SNT_Baseline | 6 | 19.9 | 40.9 | 25 | 19.5 | 43.5 | 20.3 | -13 | -5.1 | -15.2 |
| SNT_Baseline | 7 | 23 | 38 | 19.1 | 23.2 | 39.8 | 21.3 | -14.7 | -17.9 | -18.2 |
| SNT_Baseline | 8 | 20.7 | 37.1 | 14.5 | 21.5 | 38.2 | 14.9 | -10.9 | -11.7 | -22.7 |
| SNT_Baseline | 9 | 19.5 | 42 | 18.3 | 19.1 | 39.5 | 20.2 | -3.8 | -7.2 | -4.2 |
| SNT_Baseline | 10 | 20.9 | 43.8 | 20.8 | 21.1 | 41.8 | 18.1 | -1.4 | 3.2 | -10.8 |
| SNC_POD7 | 11 | 19.6 | 32.2 | 17.1 | 19.1 | 40 | 20.3 | -33.1 | -37.1 | -24.4 |
| SNC_POD7 | 12 | 19.4 | 39.6 | 21.6 | 19.1 | 42.8 | 27.9 | -20.6 | -27.5 | -16.5 |
| SNC_POD7 | 13 | 21.2 | 30.7 | 17.9 | 20.5 | 37.6 | 22.4 | -32.8 | -38.2 | -21.9 |
| SNC_POD7 | 14 | 16.6 | 37 | 19.8 | 16.2 | 40 | 22 | -19.5 | -22.3 | -14.9 |
| SNC_POD7 | 15 | 16.8 | 31.7 | 22.8 | 16.6 | 41.6 | 27.2 | -37.6 | -41.5 | -29.9 |
| SNT_POD7 | 16 | 20.5 | 35.3 | 16.7 | 18.9 | 43.5 | 20.3 | -35.2 | -39.9 | -13 |
| SNT_POD7 | 17 | 18.3 | 33.7 | 20 | 18 | 39.8 | 21.3 | -27 | -28.1 | -22.1 |
| SNT_POD7 | 18 | 21.4 | 32.1 | 19.9 | 20.6 | 38.2 | 14.9 | -23.1 | -11.3 | -19.3 |
| SNT_POD7 | 19 | 18.7 | 30.1 | 22.1 | 18.2 | 39.5 | 20.2 | -34.6 | -30.3 | -27.6 |
| SNT_POD7 | 20 | 17.3 | 34.2 | 17.2 | 17.2 | 41.8 | 18.1 | -29.6 | -30.2 | -27.1 |
| SNC_POD14 | 21 | 23.5 | 23.9 | 14.1 | 22.9 | 40 | 20.3 | -57.9 | -65.6 | -41.5 |
| SNC_POD14 | 22 | 20 | 17.9 | 14.2 | 19.5 | 42.8 | 27.9 | -79.9 | -92.7 | -56.1 |
| SNC_POD14 | 23 | 20.7 | 28.1 | 15.4 | 20.1 | 37.6 | 22.4 | -41.5 | -50.3 | -28.9 |
| SNC_POD14 | 24 | 16.9 | 26.5 | 11.1 | 17.2 | 40 | 22 | -51.8 | -66 | -43 |
| SNC_POD14 | 25 | 20 | 20.7 | 7.9 | 18.9 | 41.6 | 27.2 | -75.5 | -95.7 | -43.4 |
| SNT_POD14 | 26 | 20.7 | 23 | 10 | 20.2 | 43.5 | 20.3 | -68 | -81.9 | -47.2 |
| SNT_POD14 | 27 | 18 | 19.6 | 15.7 | 17.7 | 39.8 | 21.3 | -68.4 | -74.3 | -51.5 |
| SNT_POD14 | 28 | 20.9 | 20 | 8.6 | 20.8 | 38.2 | 14.9 | -66.7 | -77.8 | -50.4 |
| SNT_POD14 | 29 | 18.8 | 22.1 | 13.3 | 18.8 | 39.5 | 20.2 | -61.7 | -70.5 | -48.6 |
| SNT_POD14 | 30 | 20.1 | 20.1 | 13 | 19.4 | 41.8 | 18.1 | -70.8 | -77.1 | -48.8 |
| SNC_POD28 | 31 | 19.4 | 22 | 11.3 | 18.8 | 40 | 20.3 | -65.2 | -77.1 | -43.6 |
| SNC_POD28 | 32 | 25 | 21 | 14.3 | 23.7 | 42.8 | 27.9 | -73.1 | -86.2 | -44.5 |
| SNC_POD28 | 33 | 20.7 | 26.6 | 13.1 | 20 | 37.6 | 22.4 | -47.8 | -59.7 | -30.2 |
| SNC_POD28 | 34 | 19.2 | 25.7 | 14.4 | 17.9 | 40 | 22 | -55.4 | -64.6 | -29.4 |
| SNC_POD28 | 35 | 22.4 | 26.7 | 11.9 | 21.6 | 41.6 | 27.2 | -56.8 | -73.1 | -36.2 |
| SNT_POD28 | 36 | 17.7 | 20.4 | 11.5 | 17.6 | 43.5 | 20.3 | -72.9 | -84.2 | -54.8 |
| SNT_POD28 | 37 | 20.6 | 20.7 | 11.3 | 20.4 | 39.8 | 21.3 | -67.9 | -80.6 | -50.5 |
| SNT_POD28 | 38 | 16.6 | 19.8 | 9.4 | 16.1 | 38.2 | 14.9 | -67.5 | -76.8 | -46.6 |
| SNT_POD28 | 39 | 17.8 | 19.1 | 10.8 | 17.3 | 39.5 | 20.2 | -72.3 | -84.8 | -50.5 |
| SNT_POD28 | 40 | 20.4 | 14.3 | 10.5 | 20.4 | 41.8 | 18.1 | -86.5 | -96.6 | -65.8 |

**Table S3. Statistical Details by Endpoint**

| **Endpoint (Figure)** | **Group / Time point** | **n** | **Test used** | **Statistic** | **df** | **Exact p** |
| --- | --- | --- | --- | --- | --- | --- |
| Sensory recovery (Fig. 3A) | SNC vs SNT, POD28 | 5 / 5 | 2-way ANOVA, Sidak | t=6.930 | 4 | 0.0073 |
| PFI (Fig. 3B) | SNC vs SNT, POD28 | 5 / 5 | 2-way ANOVA, Sidak | t=3.965 | 32 | 0.0015 |
| TFI (Fig. 3C) | SNC vs SNT,POD28 | 5 / 5 | 2-way ANOVA, Sidak | t=1.907 | 32 | 0.2376 |
| SFI (Fig. 3D) | SNC vs SNT,POD28 | 5 / 5 | 2-way ANOVA, Sidak | t=2.496 | 32 | 0.0698 |
| ATF3 proportion (Fig. 4B) | SNC vs SNT, POD28 | 3 / 3 | 2-way ANOVA, Sidak | t=4.508 | 16 | 0.0014 |
| IB4 intensity (ipsi/con) (Fig. 5B) | SNC vs SNT, POD7 | 3 / 3 | 2-way ANOVA, Sidak | t=3.014 | 16 | 0.0325 |
| MYCN intensity (Fig. 7B) | Sham_Con vs SNC_Con, POD7 | 5 / 5 | 2-way ANOVA, Sidak | t=11.59 | 4 | 0.0047 |
| MYCN intensity (Fig. 7B) | Sham_Con vs SNC_Ipsi, POD7 | 5 / 5 | 2-way ANOVA, Sidak | t=24.52 | 4 | 0.0002 |
| MYCN intensity (Fig. 7B) | Sham_Con vs SNT_Con, POD7 | 5 / 5 | 2-way ANOVA, Sidak | t=7.99 | 4 | 0.0198 |
| MYCN intensity (Fig. 7B) | Sham_Con vs SNT_Ipsi, POD7 | 5 / 5 | 2-way ANOVA, Sidak | t=9.85 | 4 | 0.0089 |
| MYCN intensity (Fig. 7B) | SNC_Ipsi vs SNT_Ipsi, POD7 | 5 / 5 | 2-way ANOVA, Sidak | t=13.07 | 4 | 0.0030 |
| MYCN intensity (Fig. 7B) | SNC_Ipsi: POD7 vs POD14 | 5 / 5 | 2-way ANOVA, Sidak | t=21.28 | 4 | <0.0001 |
| MYCN intensity (Fig. 7B) | SNT_Ipsi: POD7 vs POD14 | 5 / 5 | 2-way ANOVA, Sidak | t=5.63 | 4 | 0.0049 |
| Iba1 intensity(ipsi/con)(Fig. 8B) | SNC vs SNT, POD14 | 3 / 3 | 2-way ANOVA, Sidak | t=3.449 | 8 | 0.0173 |
